# Draft genome sequencing of *Pediococcus pentosaceus* strains isolated from cow milk

**DOI:** 10.1128/mra.00238-24

**Published:** 2024-04-15

**Authors:** Md. Morshedur Rahman, Naim Siddique, Salma Akter, Ziban Chandra Das, M. Nazmul Hoque

**Affiliations:** 1 Molecular Biology and Bioinformatics Laboratory, Department of Gynaecology, Obstetrics and Reproductive Health, Bangabandhu Sheikh Mujibur Rahman Agricultural University (BSMRAU), Gazipur, Bangladesh; 2 Department of Microbiology, Jahangirnagar University, Savar, Dhaka, Bangladesh; University of Southern California, Los Angeles, California, USA

**Keywords:** Draft genomes, *Pediococcus pentosaceus*, Multidrug resistant, Cow milk

## Abstract

We sequenced the genomes of *Pediococcus pentosaceus* strains MBBL4 and MBBL6, isolated from raw milk samples of healthy cows. The draft genomes of the MBBL4 and MBBL6 were 1,896,831 bp and 1,849,397 bp, respectively, and were fragmented into 58 and 42 contigs, with coverages of 118.2× and 128.7×, respectively.

## ANNOUNCEMENT

*Pediococcus*, a lactic acid bacteria, is beneficial for both human and animal health (1). *Pediococcus* spp. possess the ability to produce antimicrobial peptides, commonly known as bacteriocins (2). *P. pentosaceus* defends animal health by producing antibacterial compounds like bacteriocins or bacteriocin-like substances (BLISs), preventing harmful bacteria colonization, and regulating gut microbes to protect against pathogens (3–5). Several studies showed that *P. pentosaceus* has multiple beneficial functions in human and animal health, i.e., immunomodulation by producing anti-inflammatory, antioxidant, and anti-cancer properties, as well as lowering the blood cholesterol level (6–8).

Milk samples from healthy lactating cows were collected from BSMRAU dairy farm (24.09°N, 90.41°E) located in Gazipur, Bangladesh. We conducted a California Mastitis Test to confirm the absence of mastitis in the cows (9). Milk samples were enriched on MRS broth (HiMedia, India) incubating overnight aerobically at 37°C. Subsequently, enriched samples were streaked onto MRS agar (deMan, Rogosa, and Sharpe) plates for incubation at 37°C for 48 h. We confirmed two isolates (MBBL4 and MBBL6) as *P. pentosaceus* through a VITEK-2 system v9.01 (10). Pure colonies from MRS agar were diluted in 0.45% saline to match a 0.5 McFarland standard, then inoculated into VITEK 2 system cards. After 3 h, the system identified MBBL4 and MBBL6 isolates as *P. pentosaceus* based on their biochemical properties. The isolates were subcultured in nutrient broth (Biolife, Italy) overnight at 37°C, followed by DNA extraction using QIAamp DNA Mini Kit (QIAGEN, Germany). The purity and concentration of DNA were examined using NanoDrop 2000 (Thermo Fisher Scientific, USA) (11). The Nextera DNA Flex library preparation kit (Illumina, San Diego, USA) was used to generate libraries from 1  ng DNA, and whole-genome sequencing (WGS) of the prepared libraries was performed using the Illumina MiSeq sequencer (Illumina, USA) with a 2 × 250 bp protocol. Paired-end raw reads (MBBL4 =   4,118,280 and MBBL6 = 4,216,200) underwent trimming with Trimmomatic v0.39 (12) and quality checking through FastQC v0.11.7 (13). Reads with phred scores >20 (14) were assembled using SPAdes v3.15.6 (15). Genome annotation was performed using the NCBI Prokaryotic Genome Annotation Pipeline (PGAP) v6.6 (16) with the *Pediococcus* CheckM v1.2.2 marker set (17).

The draft assembly size of MBBL4 and MBBL6 were 1,896,831 bp and 1,849,397 bp, respectively. Further sequencing and annotation statistics of both genomes are presented in Table 1. MBBL4 harbored *lnu*(A), while MBBL6 contained *lnu*(A) and *erm*(B), as detected through ResFinder 4.0 (18), indicating both isolates resistant to lincomycin. Using PHASTER (19), we predicted two prophage regions in MBBL4 and three in MBBL6 genome. PlasmidFinder (20) identified one plasmid (repUS64), and RAST server (21) predicted 199 subsystems in both genomes. BAGEL4 (22) identified one bacteriocin gene cluster (Pediocin A), while AntiSMASH v7.0 (23) detected two secondary metabolite regions in both genomes. All software was executed with default parameters, unless stated otherwise.

**TABLE 1:** Genomic features of the *P. pentosaceus* strains MBBL4 and MBBL6 isolated from raw milk of healthy cow

Strains	Genome size (bp)	Genome coverage (×)	No. of contigs	*N*_50_ value (bp)	GC content (%)	Genome completeness (%)	CheckM contamination (%)	No. of genes	Genome accession no.	SRA accession no.
MBBL4	1,896,831	118.2	58	218,362	37.3	96.08	6.5	1,938	JAZIFP000000000	SRR27558662
MBBL6	1,849,397	128.7	42	333,724	37.3	97.97	4.68	1,906	JAZIFR000000000	SRR27558660

## Data Availability

The draft genomes of *P. pentosaceus* strains MBBL4 and MBBL6 are available at NCBI/GenBank under BioProject accession PRJNA1065156. Genome accessions are available in Table 1.
